# Gene expression profile during proliferation and differentiation of rainbow trout adipocyte precursor cells

**DOI:** 10.1186/s12864-017-3728-0

**Published:** 2017-05-04

**Authors:** Marta Bou, Jerôme Montfort, Aurélie Le Cam, Cécile Rallière, Véronique Lebret, Jean-Charles Gabillard, Claudine Weil, Joaquim Gutiérrez, Pierre-Yves Rescan, Encarnación Capilla, Isabel Navarro

**Affiliations:** 1 Department of Cell Biology, Physiology and Immunology, Faculty of Biology, University of Barcelona, Av. Diagonal 643, 08028 Barcelona, Spain; 2 INRA, UR1037 Laboratory of Fish Physiology and Genomics, Campus de Beaulieu, Rennes, F-35042 France; 3 Present address: Nofima (Norwegian Institute of Food, Fisheries, and Aquaculture Research), P.O. Box 210, N-1432 Ås, Norway

**Keywords:** Adipogenesis, Teleost fish, Adipose tissue, Microarray, Transcriptome

## Abstract

**Background:**

Excessive accumulation of adipose tissue in cultured fish is an outstanding problem in aquaculture. To understand the development of adiposity, it is crucial to identify the genes which expression is associated with adipogenic differentiation. Therefore, the transcriptomic profile at different time points (days 3, 8, 15 and 21) along primary culture development of rainbow trout preadipocytes has been investigated using an Agilent trout oligo microarray.

**Results:**

Our analysis identified 4026 genes differentially expressed (fold-change >3) that were divided into two major clusters corresponding to the main phases observed during the preadipocyte culture: proliferation and differentiation. Proliferation cluster comprised 1028 genes up-regulated from days 3 to 8 of culture meanwhile the differentiation cluster was characterized by 2140 induced genes from days 15 to 21. Proliferation was characterized by enrichment in genes involved in basic cellular and metabolic processes (transcription, ribosome biogenesis, translation and protein folding), cellular remodelling and autophagy. In addition, the implication of the eicosanoid signalling pathway was highlighted during this phase. On the other hand, the terminal differentiation phase was enriched with genes involved in energy production, lipid and carbohydrate metabolism. Moreover, during this phase an enrichment in genes involved in the formation of the lipid droplets was evidenced as well as the activation of the thyroid-receptor/retinoic X receptor (TR/RXR) and the peroxisome proliferator activated receptors (PPARs) signalling pathways. The whole adipogenic process was driven by a coordinated activation of transcription factors and epigenetic modulators.

**Conclusions:**

Overall, our study demonstrates the coordinated expression of functionally related genes during proliferation and differentiation of rainbow trout adipocyte cells. Furthermore, the information generated will allow future investigations of specific genes involved in particular stages of fish adipogenesis.

**Electronic supplementary material:**

The online version of this article (doi:10.1186/s12864-017-3728-0) contains supplementary material, which is available to authorized users.

## Background

The ongoing intensification of salmonids aquaculture industry has led to the development of diets with high lipid content, which can induce an increase in subcutaneous, intramuscular and especially in visceral fat depots [1]. This excessive adiposity may entail a negative impact on the sector, reducing the efficiency of the production. Mature adipocytes are known to play an important role controlling energy balance in mammals by storing fatty acids in the form of triglycerides in periods of excess of energy and by releasing fatty acids when are needed. Despite of the relevance of this issue, very little is known about the factors regulating the development of adipose tissue in fish, and the possible health alterations associated to an increased fat deposition. While excess of adipose tissue in humans, which occurs in obesity, is known to contribute to the development of many aspects of the pathology of metabolic syndrome and other diseases [2, 3], the metabolic consequences of a high adiposity are still not well known in fish.

In order to understand the development of adiposity, it is crucial to identify the factors and mechanisms that regulate the recruitment of mesenchymal stem cells (MSCs) of the vascular stromal fraction of the adipose tissue and its transformation into lipid-filled adipocytes. Adipogenesis has been described as a two-step developmental process consisting on the commitment of undifferentiated MSCs into preadipocytes and the further development of these cells into fully functional mature adipocytes [4]. In fish, like in mammals, adipogenesis takes place as a consequence of normal cell turnover and is due to the need of storing energy [5, 6]. Therefore, growth of adipose tissue includes the hypertrophy of already existing adipocytes and the proliferation and differentiation of new ones from MSCs.

Adipogenesis has been extensively studied in mammals [7] and several cell and animal models have been successfully used to describe the chronology of the molecular events governing this process. Many are the studies highlighting the importance of the interplay of both, activating and inhibiting signals and thus involving pro-adipogenic and anti-adipogenic factors [7–10]. We have previously shown that fish primary preadipocytes differentiate into mature adipocytes in vitro and that these cells represent a very helpful model system to study adipose tissue development in fish [5, 6]. Some of the well-known key adipogenic transcription factors described in mammals, such as peroxisome proliferator activated receptor γ (PPARγ) and CCAAT-enhancer binding protein α (C/EBPα), have been identified and linked to an adipogenic function in fish as well [5, 11, 12].

Insulin favors differentiation into mature adipocytes in rainbow trout, gilthead sea bream and other fish species of cultured preadipocytes [5, 6, 13]. However, a common trait in fish is that lipids, especially high concentrations of fatty acids, play an essential role in adipocyte differentiation [5, 6, 11, 13]. However, only one study has extensively analyzed the gene expression profile during adipogenesis in Atlantic salmon [14].

Apart from controlling energy storage and mobilization, adipose tissue has an important function as a major endocrine organ secreting diverse adipokines and regulating therefore many physiological aspects [15, 16]. For instance, the cytokine tumor necrosis factor α (TNFα) is expressed in fish adipose tissue and is well known by its anti-adiposity actions [5, 17, 18]. Leptin and adiponectin are known to be the most abundant proteins secreted by mammalian adipocytes [19], contrary to what has been described so far in teleost species, where leptin is primarily expressed in the liver [20] and adiponectin in muscle [21–23]. These specific features highlight the need to further investigate the physiological and secretory functions of fish adipose tissue.

Here, to characterize the genetic basis of adipogenesis in rainbow trout, a genome-wide expression profiling combined with quantitative PCR (qPCR) and Western blot analysis was performed at 4 different time-points along the adipocyte primary culture. The main goal of this study was to provide deeper insights into the dynamics of adipocyte conversion by defining the cascades of gene expression as well as to identify possible novel adipogenic mediators and markers in this fish species. Likewise, we aimed to unravel yet undiscovered mechanisms and provide a basis for the identification of proteins that could serve in the future as potential links between the adipocyte and the process of whole body energy homeostasis.

## Methods

### Animals

Rainbow trout (*Oncorhynchus mykiss* Walbaum 1792) weighting from 200 to 250 g were obtained from the “Truites del Segre” fish farm (Lleida, Spain). Fish were acclimatized to a 12 h light:12 h dark photoperiod and 14 ± 1 °C in a closed circuit flow system located in the Faculty of Biology at the University of Barcelona for 2 weeks prior to the commencement of the experiment. Fish were fed daily *ad libitum* on a commercial diet based on fishmeal and fish oil (Dibaq Diproteg, Segovia, Spain).

### Primary culture of preadipocyte cells

All reagents were purchased from Sigma-Aldrich (Tres Cantos, Spain) and all tissue culture plastic ware was obtained from NUNC (Labclinics, Barcelona, Spain) unless stated otherwise.

Fish were fasted 24 h before the experiments. The animals were killed by a blow to the head under anesthesia (3-aminobenzoic acid ethyl ester, MS-222; 100 μg/ml), which was followed by immersion in 70% ethanol for 30 s to sterilize the external surfaces. Cells for each experiment were isolated from a pool of white adipose tissue of 6–7 fish and cultured as described elsewhere [5]. The use of pooled tissue minimizes fish-specific variation in the experimental measurements. A total of four independent cultures (*n* = 4) were performed, each of which derived from cells isolated from a different pool of white adipose tissue of 6–7 fish (i.e. 24–28 fish in total). Briefly, the dissected visceral fat tissue was washed with Krebs-HEPES buffer (pH 7.4) and was digested for 1 h with type II collagenase 130 UI/mL containing 1% bovine serum albumin in Krebs-HEPES buffer at 18 °C. The resulting cell suspension was filtered (100 μm) and centrifuged at 700 g for 10 min and the pellet was treated with erythrocyte lysing buffer (0.154 M NH_4_Cl, 10 mM KHCO_3_, and 0.1 mM EDTA) for 5 min at room temperature. After washing, the cells were centrifuged again at 700 g for 10 min. The cell pellet was resuspended in growth medium, consisting on Leibovitz’s L-15 medium containing 10% fetal bovine serum, 2 mM L-glutamine and 1% antibiotic/antimycotic solution. Cells were counted, diluted, seeded in pretreated flasks or six-well plates (25 cm^2^ or 9.6 cm^2^/well respectively), with 1% gelatin at a density of 2 · 10^4^ cells/cm^2^ and kept at 18 °C. For each experimental condition either one flask (RNA extraction) or two wells pooled together (Western blot) were used. After confluence (day 8), cells were induced to differentiate by means of a growth medium supplemented with 10 μg/mL insulin, 0.5 mM 3-isobutyl-1-methylxanthine (IBMX), 0.25 μM dexamethasone and 10 μL/mL lipid mixture. Media were changed every 2 days during the whole procedure. Samples were collected at day 3 (mesenchymal stem cells), day 8 (proliferated cells), day 15 (committed preadipocytes) and day 21 (mature adipocytes). The cells were daily assessed under light microscopy to check the morphology and ensure that they followed the already described developmental process [5, 11, 14, 24].

### RNA extraction and cDNA synthesis

Total RNA from adipocyte cells was extracted using the TriReagent method (Ambion, Alcobendas, Spain) following the manufacturer’s recommendations. The quantity of isolated RNA was determined by spectrophotometry with a ND-2000 NanoDrop (Thermo Fisher Scientific, Alcobendas, Spain) and the quality was assessed using a Bionalyzer (Agilent). The total RNA was used for microarray and qPCR analysis.

For cDNA synthesis 5 μg of total RNA was reverse-transcribed using High Capacity cDNA Reverse Transcription Kit (Applied Biosystems) in a 25 μl reaction volume according to the instructions provided by the manufacturer. The reverse-transcription products (cDNA) were diluted by the addition of nuclease-free water to a final volume of 200 μl. Reverse transcription was performed in duplicate for each individual sample.

### Quantitative real-time PCR (qPCR) analysis

qPCR measurements were performed in a StepOnePlus Tm system (Applied Biosystems) as described in C Weil, V Lebret and JC Gabillard [25]. Briefly, 5 μl of diluted cDNA was amplified in duplicates containing Fast SYBR Green Master Mix (Applied Biosystems) and specific primers at a final concentration of 600 nM. The qPCR primer sequences for the target genes and the reference gene are shown in Table 1. Primers were designed to span exon-boundaries if possible (Genamics Expression software) using known sequences from the rainbow trout nucleotide databases (INRA-Sigenae) and previously published [25, 26].

**Table 1:** Rainbow trout primer sequences used for qPCR

Gene	Accession number	Primer sequence (5'-3')	Annealing T (°C)
ACSL1	CR363150.p.om.8	**F:** TGCAATCTAGCAAGGTTCCTTTTG	60
		**R:** TCCAAGCAGAAACCCAGTACAGAA	
GPDH	AF027130.p.om.8	**F:** ATGACCACAGTCCACGCCTACAC	60
		**R:** GGCAGGTTAGGTCCACCACTGA	
IGFBP5	DQ206713	**F:** ACTTCACGCGCTTCTCCATGGCA	60
		**R:** CGAGACTCATGATCTATGGGTGGA	
IGFBP7	DQ146965	**F:** GCTCCGATGGAGTGACCTATA	60
		**R:** ACAATGACAGGTGCTGTTGCG	
PCNA	CA358086.s.om.10	**F:** ATACGGGCAAACTCTCCTGATGGC	60
		**R:** CAACGCAGACACACTCGCCCTT	
cPEPCK	[26]	**F:** CCCAGTGCCTGTGGGAAAAC	60
		**R:** CCACACCGAAAAAGCCGTTC	
PLIN2	CB494091.p.om.8	**F:** GATGGCAATGAGGCAGAGAACA	60
		**R:** AGGCAGAGTGGCTAAGGGACAG	
18S	AF308535	**F:** CGGAGGTTCGAAGACGATCA	60
		**R:** TCGCTAGTTGGCATCGTTTAT	

F, forward; R, rever se; T, temperature

Relative quantity of each gene was determined from a standard curve consisting of serial dilutions of a pool of cDNAs obtained from isolated mature adipocytes and expressed as Arbitrary Units (A.U.). This calculation was performed using the software included in the StepOne plus Tm System which corrects for differences in amplification efficiencies. Primer efficiencies ranged from 90 to 103%. Specificity of the amplification reaction was verified by analysis of melting curves. 18S was used for the normalization of qPCR data since its RNA abundance was stable along the cell culture (p > 0.05). The two cDNAs obtained from each sample were run in a single plate for each gene assay together with negative controls (reverse transcriptase-free samples and RNA-free samples).

### Microarray analysis

The adipocyte transcriptome was analyzed using an Agilent-based microarray platform with 8 x 60 K probes per slide previously described [27] and registered in GEO with the platform record GPL15840. Microarray data sets have been submitted to the GEO-NCBI with the accession number GSE90058. Total RNA from cells at different developmental stages (days 3, 8, 15 and 21) from four independent cultures, each of which derived from cells isolated from a different pool of white adipose tissue of 6–7 fish, was labelled with Cy3 according to the manufacturer’s instructions (Agilent). Briefly, RNA was first reverse transcribed, using a polyDTT7 primer, Cy3 was incorporated by a T7 polymerase mediated transcription and excess dye was removed using an RNeasy kit (Quiagen). The level of dye incorporation was evaluated using a spectrophotometer (Nanodrop ND1000, LabTech). Labelled RNA was then fragmented in the appropriate buffer (Agilent) for 30 min at 60 °C before dilution (v/v) in hybridization buffer. Hybridizations were performed in a microarray hybridization oven (Agilent) overnight at 65 °C, using two high-density oligonucleotide microarray slides. Following hybridization, the slides were rinsed in gene expression wash buffers 1 and 2 (Agilent) and scanned at a 3 μm resolution using an Agilent G2505 microscanner. Fluorescence intensity was calculated using the standard procedures contained in the Agilent Feature Extraction software version 10.7 and the data were normalized using GeneSpring software. An ANOVA (*p* < 0.02) and an average fold change of >3 were used as the criteria for defining genes as differentially expressed between the different days along the cell culture. For clustering analysis, data were log transformed, median-centered and an average linkage clustering was carried out using CLUSTER software. The results were visualized using TREEVIEW [28]. Biological functions and pathways were generated and analyzed using Ingenuity Pathway Analysis software (IPA, Ingenuity Systems, CA).

### Western blot analysis

Protein extraction and Western blot analysis were performed as previously reported [29]. Twenty μg of protein were subjected to SDS-PAGE gel electrophoresis and Western blot analysis was performed using the appropriate antibodies for long-chain acyl-CoA synthetase (ACSL-1) (cat no 98925), proliferating cell nuclear antigen (PCNA) (cat no 7907), phosphoenolpyruvate carboxykinase (PEPCK) (cat no 32879), perilipin 2 (PLIN2) (cat no 32888), all from Cell Signaling Technology Inc (Beverly, MA) and PPARγ (PPARγ; cat no 7196; Santa Cruz Biotechnology). Immunoreactive bands were visualized using an enhanced chemiluminescence kit (Pierce ECL Western Blotting Substrate; Thermo Scientific, Alcobendas, Spain) and quantified by densitometric scanning using ImageJ software (National Institutes of Health, Bethesda, MD, USA). Some of these antibodies have been previously shown to successfully cross-react with fish, such as PPARγ [5] and PCNA [30]. For those antibodies not tested previously, homogenates of rat liver were also run as a control in a contiguous lane to confirm the specificity of immunodetection (i.e. apparent molecular weight of the band). Western blots results were obtained from four independent cultures, each of which derived from cells isolated from a different pool of white adipose tissue of 6–7 fish.

## Results and discussion

### Gene expression profile during adipogenesis

**Fig. 1:**
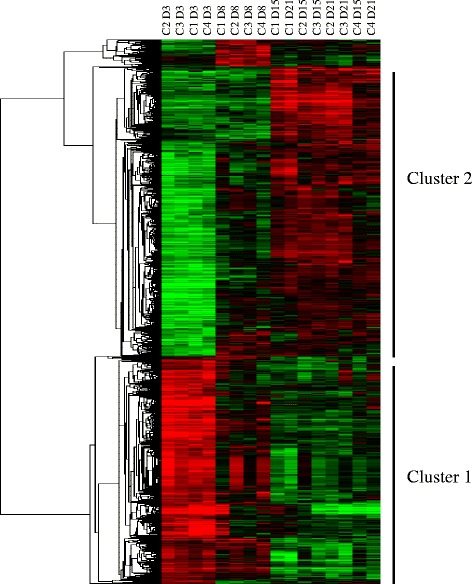
Heat map of the hierarchical clustering of genes differentially expressed (>3.0-fold, *p* < 0.02) among different days along rainbow trout adipocyte culture. The horizontal dendrogram represents the correlation distances between gene expression levels. Every column represents one independent culture (C) at one time-point (D3, D8, D15; D21; days 3, 8, 15, 21 respectively) meanwhile the different genes are represented by individual rows. The *red* color indicates high levels of expression while green represents low levels of expression. The intensity of both colors is related to the transcript expression level. Data are presented in the same format in Figures 2-8

In order to characterize the process of adipogenesis in rainbow trout, four independent primary cultures of adipocytes were performed and samples were collected at days 3, 8, 15 and 21 post seeding and used for microarray experiments. After bioinformatics analysis a total of 4026 genes were found differentially expressed (>3.0-fold, *p* < 0.02). Hierarchical clustering analysis of these differentially expressed genes was performed and two major clusters were identified (Fig. 1) and related to the main phases described during preadipocyte culture; proliferation (cluster 1) and differentiation (cluster 2). Proliferation cluster comprised 1028 genes that were up-regulated from days 3 to 8 of culture meanwhile the cluster characterizing the differentiation phase included 2140 up-regulated genes from days 15 to 21 of culture.

### Functional annotation of genes found in cluster 1 (Proliferation) and 2 (Differentiation)

Cluster 1 comprised genes that were up-regulated during days 3 and/or 8 of culture. Significant biological functions associated with the proliferative phase of adipogenesis were determined by using Ingenuity pathway analysis (IPA) software (Additional files 1 and 2). The early stages of adipocyte development were characterized by a large number of genes involved in basic cellular and metabolic processes such as transcription, ribosome biogenesis, translation and protein folding. During this part of the culture the category of “Cellular Growth and Proliferation” was highly represented, with 254 molecules included in the functional annotation of “proliferation of cells” (*P* = 2.10E^−16^). Among the other significant categories present in the early stages of adipocyte development, the category “Tissue Morphology” that included the functional annotations of “quantity of cells” (*P* = 1.41E^−17^) and “morphology of connective tissue” (*P* = 1.02E^−6^) and the category of “Immune Cell Trafficking” (*P* = 5.43E^−14^ – 3.07E^−04^) and “Inflammatory Response” (*P* = 1.40E^−12^ – 3.16E^−04^) were also found. On the other hand, the “Eicosanoid Signaling” (*P* = 1.5E^−04^) and the “Interleukin 8 (IL-8) Signaling” (*P* = 4.73E^−04^) pathways were found within the top canonical pathways.

On the other hand, cluster 2 consisted on up-regulated genes during days 15 and/or 21 of culture. During this second phase a remarkable number of genes involved in energy production, glucose and lipid metabolism were revealed by IPA analyses (Additional files 3 and 4). IPA analysis highlighted as top categories “Molecular and Cellular Functions” and “Molecular Transport”, which included the functional annotations “transport of molecules” (*P* = 8.05E^−11^), “concentration of lipid” (*P* = 1.82E^−9^) and “transport of lipid” (*P* = 4.22E^−7^) among others. Other top category represented was “Carbohydrate Metabolism”, comprising 128 molecules and including functional annotations such as “glycolysis of cells” (*P* = 1.33E^−10^), “metabolism of carbohydrate” (*P* = 3.97E^−9^) and “gluconeogenesis” (*P* = 8.80E^−5^). The category of “Cellular Growth and Proliferation” was also highly represented, with 250 molecules included in the functional annotations of “proliferation of cells” (*P* = 1.28E^−11^) and “proliferation of connective tissue cells” (*P* = 3.35E^−8^). “IL-8 signaling” (*P* = 2.58E^−5^) was present again as one of the top canonical pathways, together with “TR/RXR Activation” (*P* = 1.15E^−7^). Finally, the implication of the PPAR signaling pathway during this phase of the culture was also revealed (*P* = 1.37E^−7^).

In order to describe and highlight the different processes that take place during culture development, including the proliferation and differentiation phases, the following results and corresponding discussion will be divided into different sections according to some selected functional groups of molecules. The first section will cover the main processes present during the early stages of adipocyte commitment and development. Next, the coordinated cascade of transcription factors that orchestrate fat cell progression will be presented, followed by the description of the main epigenetic factors identified. Subsequently, the relevance of two regulatory systems, the insulin and insulin-like growth factors (IGFs) and the eicosanoid signaling, will be discussed. Finally, a section concerning the main terminal regulators of adipocyte maturation present in our model system will be provided.

### Cell commitment and remodelling

**Fig. 2:**
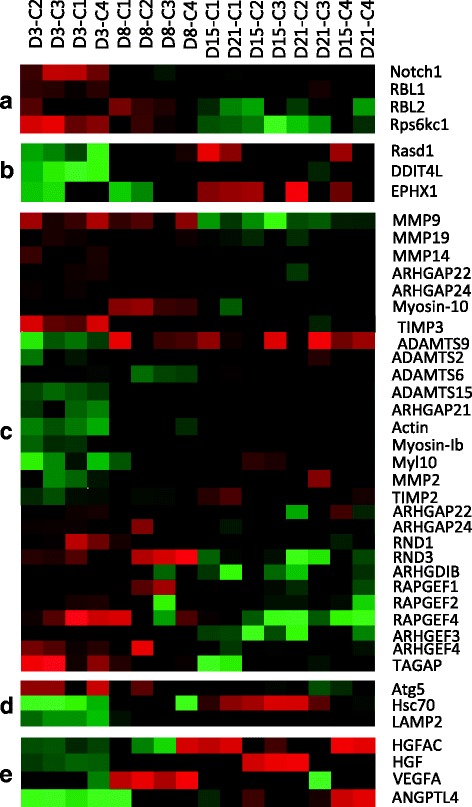
Selected genes involved in cell commitment and remodelling. **a** Cell commitment; **b** Adipogenic markers; **c** Cell remodelling; **d** Autophagy; **e** Angiogenesis

Initial phases of adipogenesis are characterized by the presence of pluripotent precursors (i.e. MSCs) of adipocytes. Many genes involved in cell fate (Fig. 2a) were differentially induced in the early steps of our cell culture. For instance, the presence of Notch1, which is a protein responsible for cell fate decision and implicated in the regulation of adipogenesis in 3T3-L1 cells [31], was revealed during the proliferative phase of the culture. During this phase we found overexpressed also the ribosomal protein S6 kinase delta-1 (Rps6kc1 or S6K1). S6K1 is involved in the commitment of embryonic stem cells to early adipocyte progenitors in mice [32] and, is known to promote protein synthesis, cell growth and cell proliferation; processes that are of utmost importance during the early stages of the culture. On the other hand, the expression of the retinoblastoma-like proteins 1 (RBL1/p107) and 2 (RBL2/p130), which control cell cycle progression, was also evident during the first stages of cell development. RBL1 has been reported to be a crucial regulator for determining the adipocyte fate choices of stem cells committing to the white lineage, whereas its suppression is required for commitment to the brown-type fate [33]. In addition, alterations in the expression of both, RBL1 and RBL2 inhibit adipocyte differentiation [7]. In this sense, their expression in early stages and their down-regulation during days 15 and 21 of our culture will support this observation suggesting a role for these molecules in adipocyte commitment also in rainbow trout.

Some of the adipogenic markers described in human bone marrow-derived MSCs differentiating into the adipogenic lineage were present in our transcriptome study. Such is the case of dexamethasone-induced Ras-related protein 1 (RASD1), DNA damage-inducible transcript 4-like protein (DDIT4L) and epoxide hydrolase 1 (EPHX1), which are highly expressed during adipocyte development with a peak at day 15 of culture (Fig. 2b) [34].

The dramatic alteration in cell shape, from a fibroblast to a spherical shape represents one of the first hallmarks of adipogenesis. Our analysis has shown the regulation of a large number of genes involved in this type of processes (Fig. 2c). For instance, we found a remarkable presence of members of the matrix metalloproteinases (MMPs) family, as well as transcripts encoding their tissue inhibitors (TIMPs), which are known to regulate MMP activity all along the culture [35]. MMP9 was up-regulated during the proliferative phase while MMP2 was increased during differentiation. In freshly isolated mature adipocytes from humans both transcripts are present, and in agreement with our data, the expression of MMP2 was higher than that of MMP9. Moreover, in the same line, the expression analysis of MMPs during 3T3-F442A preadipocyte differentiation revealed that MMP2 showed a 13-fold increase in mRNA levels after 9 days of differentiation compared with non-differentiated cells, while MMP9 followed only a moderate increase during differentiation, reaching a maximal level after 7 days, and decreasing afterwards [36]. Accordingly, in the same study the inhibition of MMP2 and MMP9 markedly decreased adipocyte differentiation, highlighting their role during the development of these cells [36]. Furthermore, we also found MMP14 up-regulated during proliferation. The loss of this protein impairs adipogenesis in vivo in mice [37]. The relevance of the proteolytic activity during rainbow trout adipogenesis was also evidenced by the presence of many transcripts encoding various members of the ADAMTS (a disintegrin and metalloproteinase domain, with thrombospondin motifs) family during the last stages of development (days 15 and 21). Therefore, it seems that the balance and coordination among these factors might have a prominent role during both, early and late phases of adipocyte development.

In addition, cell shape and extracellular matrix remodeling have been found to regulate preadipocyte commitment and competency by modulating among others the RHO-family GTPase signaling cascade in mammals [38]. Our data suggests that this could also be the case for rainbow trout adipocytes, due to the high number of genes of this family especially up-regulated during the early steps of the process. In this sense, cluster 1 was enriched with many representatives, such as Rho GTPase activating protein 22 (ARHGAP22) and 24 (ARHGAP24), Rho family GTPase 1 (RND1) and 3 (RND3), Rho GDP dissociation inhibitor beta (ARHGDIB), Rap guanine nucleotide exchange factor 1 (RAPGEF1), 2 (RAPGEF2) and 4 (RAPGEF4), Rho guanine nucleotide exchange factor 3 (ARHGEF3) and 4 (ARHGEF4) and T-cell activation RhoGTPase activating protein (TAGAP). Actin and myosin are examples of key molecules involved in cell shape mediated differentiation that are on the contrary up-regulated in late developmental phases [39].

In line with the dramatic cytoplasmic reorganization that occurs during adipogenesis, we found in the proliferation cluster the presence of Atg5, a key autophagy gene. Autophagy is a major cytoplasmic degradation pathway that has been linked to the regulation of adipogenesis in mammals [40]. It does not only participate in cellular remodelling, but it also plays an important role controlling the dynamic change in mitochondrial mass that takes place during the maturation process [41]. Loss of Atg5 results in impaired white adipose tissue development both, in vitro and in vivo in mice [42, 43]. In addition, transcripts encoding heat shock cognate protein of 70 kDa (Hsc70) and lysosomal-associated membrane protein type 2A (LAMP2A), relevant proteins involved in chaperone-mediated autophagy [44], were increased in the cluster representing the differentiation phase, indicating that autophagy might be playing an important role during the whole differentiation process (Fig. 2d).

In humans, adipose-derived stem cells have been defined as one of the most promising stem cell types for its use in cell-based therapies to treat diverse diseases. This is due to the number of angiogenic and anti-apoptotic growth factors that these cells secrete at bioactive levels. Adipogenesis and angiogenesis are known to be tightly associated in mammals [45], being angiogenesis required for adipose tissue expansion [46]. The angiogenic capacity of our cell system is early displayed (from day 8) (Fig. 2e), with the up-regulation of transcripts encoding some of the most relevant genes involved in this process, such as the hepatocyte growth factor (HGF), the vascular endothelial growth factor A (VEGFA) and the angiopoietin-related protein 4 (ANGPTL4). This suggests that, as in mammalian adipose-derived stem cells, which are reported to be involved in the regeneration of the ischemic myocardium [47], rainbow trout adipocytes may have also an active role in the regeneration of tissues.

### Coordinated expression of transcriptional factors during adipocyte differentiation

#### AP-1 complex

**Fig. 3:**
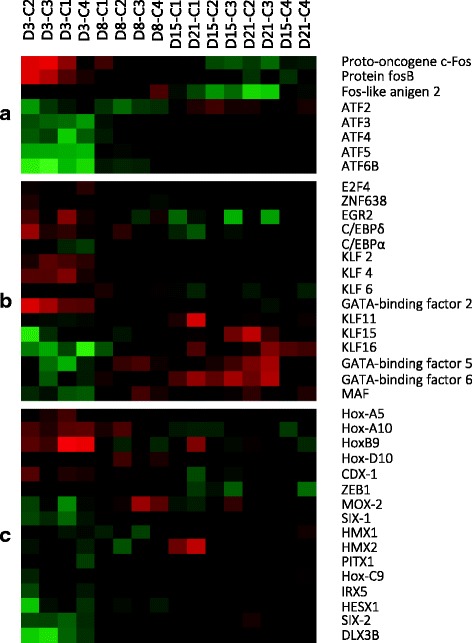
Selected transcriptional regulators overexpressed during rainbow trout adipogenesis. **a** AP-1 complex; **b** E2F, C/EBP, Krüppel-like factor and GATA families; **c** Homeobox containing transcriptional factors

A large number of transcription factors were up-regulated with different temporal expression patterns over the course of rainbow trout adipocyte differentiation. Experiments using different cellular model systems have described the existence of a tightly regulated cascade of transcription factors that promote the differentiation of fat cells [4]. In white adipocytes this cascade starts with the activation of members of the activator protein-1 (AP-1) transcription factor complex [48]. AP-1 consists of dimers of proteins belonging to the Fos, Jun and activating transcription factor (ATF) families that have been reported to present different functional properties [49]. AP-1 works as an environmental biosensor of the cell, regulating different aspects of cell physiology in response to stress or growth factors [50]. During the first period of rainbow trout adipocyte development (Fig. 3a) we found the presence of c-Fos, which has been described as essential to initiate adipocyte differentiation and identified as part of a nuclear complex that regulates the expression of adipocyte-specific genes [51]. More recently, the ongoing differentiation process in 3T3-L1 cells was inhibited by the knockdown of this transcription factor [52], and in humans, a mutation in this gene has been associated with the development of congenital generalized lipodystrophy [53]. We also found the transcription factor FOS-like antigen 2 (FOSL2, also referred to as Fra2) highly expressed during the first stages of cell development (Cluster 1). Even though this transcription factor is not considered as adipocyte-specific, it has been described to promote the expression of the adipokine leptin in both, human and mouse adipocytes [54]. In addition, its implication in adipocyte homeostasis has been evidenced since its deletion triggers a high adipocyte turnover able to regulate body fat mass in mice [55]. On the other hand, other transcription factor from the AP-1 complex, the protein FosB was also up-regulated during the same period. The overexpression of this gene has been reported to provoke inhibition of adipogenesis in mice and is suggested to exert this action through an interaction with C/EBPβ [56]. Interestingly, several members of the ATF family (ATF2, ATF3, ATF4, ATF5, and ATF6B) were found overexpressed only during the differentiation phase of the rainbow trout culture. Many of these genes are known to mediate cellular stress response signalling; and for instance ATF3 has been recently involved in adipocyte differentiation although it appears to have an inhibitory action, suggesting that a coordinated equilibrium between members of the different families that conform the AP-1 complex would be necessary [57]. Overall, the parallelism of gene expression profiles in mammalian models and the present study in AP-1 components suggests a similar role of this transcription factor complex in fish, with some species-specific responses. In this sense, a different temporal activation of this complex was reported in Atlantic salmon adipocytes, where the induction of AP-1 was observed during the last stages of adipogenesis [14].

#### E2F, C/EBP, Krüppel-like factor (KLF) and GATA families

The E2F family of transcription factors is known to regulate cell cycle progression [7] as well as adipocyte differentiation [58]. In our study we found E2F4 highly expressed at day 3 of the culture, and then the expression went down (Fig. 3b). In mammals, E2F4 forms a complex with RBL2 that is able to repress the transcription of PPARγ, and then is lost after hormonal stimulation to allow adipogenesis to proceed [59]. We observed that E2F4 and RBL2 follow the same expression pattern during rainbow trout adipocyte development (Figs. 2a and 3b), suggesting that their down-regulation might be required to promote adipogenesis also in this species. Likewise, the recently reported novel regulator of pro-adipogenic genes, zinc finger protein 638 (ZNF638) [60] was induced early during rainbow trout adipocyte development, presenting its peak of expression at day 3 as well as the early growth response protein 2 (EGR2, also known as KROX20), which has been described as a factor capable of inducing uncommitted fibroblasts to become adipocytes [61].

During rainbow trout adipogenesis we also identified a transient induction of several members of the C/EBP family of transcription factors (Fig. 3b). C/EBPδ, which is a key early regulator of adipogenesis, presented its peak of expression at day 3 of cell development. C/EBPδ together with C/EBPβ, have been reported to transactivate CEBPα [62]. In our cell system, CEBPα started to be up-regulated from day 8, with a maximum peak at day 15. This transcription factor, together with PPARγ, is known to coordinate and induce the expression of several genes involved in insulin sensitivity, lipogenesis and lipolysis promoting the adipocyte phenotype [63]. Immunofluorescence results showed a high presence of both transcription factors, CEBPα and PPARγ, in rainbow trout differentiating adipocytes at day 11, suggesting their implication in the regulation of these processes [5], as supported now by the present results.

Several members of the multigenic Krüppel-like factor (KLF) family have been reported to control adipogenesis, some being considered activators (KLFs 4, 5, 6 and 15) while others are considered repressors of transcription (KLFs 2 and 7) [48, 63]. During the progression of our culture we observed a sequential expression of members of this family (Fig. 3b). Thus, during the early stages of cell development we identified transcripts encoding the KLFs 2, 4 and 6, while transcripts encoding the KLFs 11, 15 and 16 were present during adipocyte maturation. Some of them have been extensively studied in the context of adipocyte progression. For instance, KLF4 has been described as an essential early activator of adipogenesis able to bind to Krox20 to transactivate C/EBPβ [64]. KLF15 is known to play an important role in adipocyte insulin sensitivity through the transactivation of the facilitative glucose transporter (GLUT4) promoter by binding near the myocyte enhancer binding factor-2A (MEF2A) consensus site [65]. Less is known about the roles of this family of transcription factors in fish. In Fugu, a potential binding site for KLF15 and MEF2 has been identified also in the GLUT4 promoter region, indicating a remarkable conservation among vertebrates [66]. In zebrafish, several members have been identified and characterized, and a high degree of functional conservation with the mammalian homologs was reported [67] with a special focus on the function of these factors in hematopoiesis [68]. On the other hand, an important role of this family of proteins in rainbow trout oogenesis has been suggested, being this a conserved feature among vertebrates [69]. Therefore, and in light of our transcriptomic data, we could speculate that the KLF family plays a critical function during rainbow trout adipogenesis with some of their actions maintained during evolution from fish to mammals.

GATA factors are generally considered as negative regulators of adipogenesis. We found GATA2 up-regulated during proliferation (Fig. 3b), with a significant peak of expression at day 3. This is in agreement with previous findings reported in the mammalian system, where the expression of this transcription factor is restricted to the preadipocyte phase and is down-regulated upon adipocyte differentiation [70]. Two other members from this family were significantly expressed during rainbow trout adipogenesis with different expression patterns, GATA5 and GATA6 (Fig. 3b); however, to the best of our knowledge the implication of these members in adipogenesis has not been reported so far.

As previously mentioned, adipocyte development is regulated by a network of multiple transcription factors, and V-maf avian musculoaponeurotic fibrosarcoma oncogene homolog (MAF) is likely to be involved in coordination with other factors [71]. The expression of MAF has been reported to increase during human adipogenesis [72] and this seems to be the case in rainbow trout culture, since its transcript abundance was increased from day 8 post-seeding.

#### Homeobox genes

Furthermore, with regards to other transcription factors involved in adipogenic differentiation, we also found a large number of transcripts encoding homeobox-containing genes that were differentially regulated (Fig. 3c). During the early stages of adipocyte development several Hox family genes, such as HoxA5, HoxA10, HoxB9 and HoxD10 were up-regulated. Hox genes are responsible for embryonic and adult development [73] and even though they have been reported to be involved in the adipogenic process of different model systems [74, 75], their expression pattern differs depending on the localization of fat depots within the human body [76]. Transcripts from four other homeobox-containing factors were present during the proliferative phase; mesenchyme homeobox 2 (MEOX2), SIX homeobox 1 (SIX1), caudal type homeobox 1 (CDX1) and zinc finger E-box-binding homeobox 1 (ZEB1). Interestingly, ZEB1 has been recently described as a key transcriptional component in the regulation of mouse pre-adipocytes development among other functions [77]. The second phase of rainbow trout adipogenesis was also characterized by the presence of many transcripts belonging to these groups of proteins. Among them we found up-regulated in the differentiation cluster: H6 family homeobox 1 (HMX1) and 2 (HMX2), paired-like homeodomain 1 (PITX1), homeobox C9 (HoxC9), iroquois homeobox 5 (IRX5), HESX homeobox 1 (HESX1), SIX homeobox 2 (SIX2) and distal-less homeobox 3b (DLX3B). The implication of homeobox transcription factors as relevant players in the regulation of adipose tissue functions have been recently reported [78]. The strong enrichment of these kinds of genes during rainbow trout adipogenesis could therefore indicate that they are also relevant molecules regulating adipose tissue development in fish.

All in all, a sequential activation of different transcription factors is present during the transformation of precursor cells into fully differentiated mature adipocytes in rainbow trout. Moreover, the general progression found seems to be very similar to that already described in different mammalian models, although functional studies of some of these factors would reveal possible fish-specific roles during adipogenesis.

### Epigenetic factors during adipogenesis

The dynamic remodeling of chromatin and its influence on adipogenic gene expression has been previously reported in other cell systems [79–81]. Histone modifications are relevant mechanisms to study since they are known to modulate the transcriptional regulation of factors that govern adipogenesis. However, these kinds of processes have not been explored in fish adipocytes so far.

**Fig. 4:**
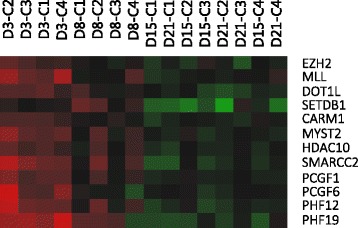
Epigenetic transcriptional regulators overexpressed during the proliferative phase of rainbow trout adipocyte development

Our results suggest that there is an important link between transcriptional regulation and epigenetic modulation. We have found a large number of up-regulated genes encoding epigenetic transcriptional regulators, especially during the proliferation phase (Fig. 4). We detected several histone-lysine N-methyltransferases, such as enhancer of zeste 2 polycomb repressive complex 2 subunit (EZH2), lysine (K)-specific methyltransferase 2A (MLL), DOT1-like histone H3K79 methyltransferase (DOT1L) and SET domain, bifurcated 1 (SETDB1). Surprisingly, SETDB1 is one of the methyltransferases known to inhibit adipogenesis by repressing PPARγ [82]. On the contrary, EZH2 has been described to present pro-adipogenic activity, since it silences Wnt genes, which are known to be negative regulators of adipogenesis [83]. Other genes coding for proteins involved in chromatin remodeling during the first stages of adipocyte development are the histone-arginine methyltransferase CARM1, the histone acetyltransferase MYST2 and the histone deacetylase 10 (HDAC10), SWI/SNF related, matrix associated, actin dependent regulator of chromatin, subfamily c, member 2 (SMARCC2), and members of the Polycomb group (PcG) of proteins such as PCGF1, PCGF6, PHF12 and PHF19.

### Specific regulatory systems

#### Insulin and IGF system

**Fig. 5:**
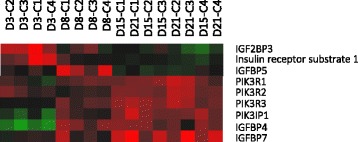
Selected genes involved in the insulin and IGF system overexpressed during rainbow trout adipogenesis

The insulin and IGFs system plays a critical role in mammalian adipocyte models. Insulin, IGF-I and IGF-II expression did not change along the rainbow trout cell culture; however, differential patterns of expression were observed for several IGF binding proteins (IGFBPs). IGF 2 mRNA-binding protein 3 (IGF2BP3), which has been described as a translational activator of IGF-II and further leads to the activation of its immediate downstream effectors promoting proliferation and angiogenesis [84] was up-regulated during the proliferation phase (Fig. 5). IGFBP4 and IGFBP7 were up-regulated during differentiation, while IGFBP5 decreased (Fig. 5). IGFBPs can inhibit and/or potentiate IGF actions, depending on the cellular context and experimental conditions [85]. In vascular smooth muscle cells, when added together with IGF-I, IGFBP4 exerts an inhibitory effect on IGF-I-induced DNA synthesis, while IGFBP5 potentiates the mitogenic effect of IGF-I, which is in concordance with the expression profile of IGFBPs observed in our study. Besides, some IGFBPs such as IGFBP3 and IGFBP5 have been also shown to have intrinsic biological activities that are IGF-independent [86, 87]. In this sense, IGFBP3 inhibits both 3T3-L1 preadipocyte differentiation initiated by insulin as well as insulin action in differentiated adipocytes. Although no changes in IGFBP3 were observed in our cells, a decline in IGFBP5 was found during the differentiation phase as it has been reported in porcine cells [88].

Moreover, insulin and IGF-I receptors did not show significant variations along the culture but regarding its signaling pathway, insulin receptor substrate 1 (IRS1) was up-regulated during proliferation (Fig. 5). IRS-1 and IRS-2 have multiple tyrosine residues, which are used as ‘docking’ sites for downstream signaling molecules and are linked to differential processes [89]. IRS-1 mediates both metabolic and proliferative effects of IGF-I, but its mitogenic actions are more relevant [89, 90], which agrees with its higher expression during the proliferative phase of our culture compared to the differentiation phase. Several genetically altered mouse models of IGF-I signaling provide clues to understanding the role of this growth factor in adipogenesis. A point mutation in the IRS-1 gene induced growth retardation and reduced the amount of adipose tissue in mice [91].

Key components of the insulin and IGF-I signaling cascade such as phosphatidylinositol 3-kinase regulatory subunits alpha (PIK3R1), beta (PIK3R2), gamma (PIK3R3) and phosphoinositide-3-kinase-interacting protein 1 (PIK3IP1) were up-regulated during differentiation (Fig. 5). This regulation agrees with the fact that this pathway activates metabolic processes, abundant during adipocyte maturation in these later phases being for instance key signaling molecules in the stimulation of glucose uptake. Human preadipocytes, as is the case for murine preadipocytes, depend on it to undergo complete differentiation [92].

#### Eicosanoid metabolism

**Fig. 6:**
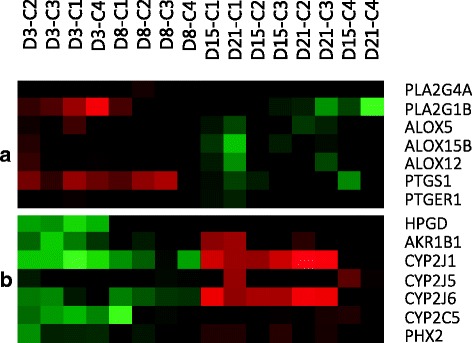
Selected genes involved in eicosanoid metabolism. **a** Genes overexpressed during the proliferative phase; **b** Genes overexpressed during the differentiation phase

Arachidonic acid (AA) is a polyunsaturated omega-6 fatty acid that is released from the membrane phospholipids by the activity of the cytosolic phospholipase A2 (cPLA2). Then, free AA can be metabolized to eicosanoids through three major routes: the lipoxygenase (LOX), the cyclooxygenase (COX) and the cytochrome P450 (CYP) pathways. These signalling pathways are relevant for adipocyte development, since some of its products have been reported to activate lipid biosynthesis during the acquisition of the adipocyte phenotype [93] and are implicated in other functions related to immune response and inflammation. In line with this, the relevance of the activation of different immune pathways during Atlantic salmon adipogenesis has been previously reported [14]. During the early steps of adipocyte culture, we found up-regulated different genes encoding members of the PLA2 class of enzymes (Fig. 6a), such as PLA2G4A and PLA2G1B, pointing out to the early implication of eicosanoid metabolism in the process of rainbow trout adipocyte development. This is in agreement with the results from the functional analysis performed, where the eicosanoid signaling pathway was highlighted during the proliferative phase of the culture. In mammals, eicosanoids are known to serve as natural ligands to activate PPARs [94]; whether fish adipocytes activate the eicosanoid metabolic pathway to also regulate PPARγ remains unknown.

A critical function of LOX activity in the early steps of mammalian adipocyte development has been reported, since there are enough evidences supporting that LOX-derived endogenous products might be also ligands involved in the transactivation of PPARγ [95]. Interestingly, and in agreement with these findings, the search of LOX genes in the microarray data only presented results during the proliferative phase. In the transcriptome data we found an up-regulation of the genes arachidonate lipoxygenases ALOX5, ALOX15b and ALOX12 during days 3 and 8 of the culture (Fig. 6a); all of which have been reported to be implicated in mammalian development of the adipocyte [96].

AA metabolites from the COX pathway formed by 3T3-L1 adipocytes have been reported to regulate the progression of adipogenesis through an autocrine control mechanism [97, 98]. The identification of several genes involved in the COX pathway exhibiting significant changes in expression during rainbow trout adipogenesis is stressing the possible implication of this pathway in the adipocyte maturation process. Thus, in the cluster associated with proliferation we found up-regulated prostaglandin G/H synthase 1 (PTGS1; also known as COX1) and prostaglandin E2 receptor EP1 subtype (PTGER1) (Fig. 6a). During the differentiation phase other genes implicated in the metabolism of prostaglandins were present, such as 15-hydroxyprostaglandin dehydrogenase [NAD+] (HPGD) and the aldose reductase AKR1B1 (Fig. 6b), as it was recently shown in human multipotent adipose-derived stem cells [99].

In the CYP pathway, AA is converted to epoxyeicosatrienoic acids (EETs) and 20-HETE by CYP epoxygenases and CYP ω-hydroxylases, respectively [100]. EET biosynthesis can be accomplished by different P-450 isozymes, being the members of the CYP2J and CYP2C epoxygenases of especial relevance in this process. Then, EETs are rapidly hydrolyzed by soluble epoxide hydrolase (EPHX2) to form less active dihydroxyeicosatrienoic acids (DHETs). In this sense, the microarray data revealed a remarkable presence of different members of these genes overexpressed during the differentiation phase such as CYP2J1, CYP2J5, CYP2J6, CYP2C5 and EPHX2 among many others (Fig. 6b). A dysregulation of the CYP epoxygenase pathway has been recently reported to be a pathological consequence of obesity [101], emphasizing the importance of the bioavailability of EETs for a healthy and functional adipose tissue.

### Terminal differentiation regulators

As soon as the cells start to present an adipocyte-like phenotype (days 15 and 21), a remarkable number of genes critical for many aspects of carbohydrate and lipid metabolism were overexpressed in comparison with previous stages (days 3 and 8). Regarding the activation of the carbohydrate metabolism, we identified and selected for further validation the gene encoding one of the main enzymes controlling gluconeogenesis; phosphoenolpyruvate carboxykinase 1 (PEPCK). Some other selected genes were also tested by qPCR (see Fig. 9). The development of one of the main functions of adipocytes, which is its high capacity to store energy, was highlighted by the large presence of genes involved in metabolic trapping, glycogen synthesis, the pentose phosphate pathway, glycolysis, oxidative phosphorylation and all aspects of lipogenesis. On the other hand, the differentiation phase was also characterized by up-regulation of genes required for regulating the mobilization of stored energy, corroborating one of the basic roles of adipocytes, being a buffer tissue storing and providing energy depending on body demands.

Alongside the development of the machinery involved in lipid and carbohydrate metabolism, the activation of the thyroid-receptor/retinoic X receptor (TR/RXR) and the PPAR signalling pathways was observed during the differentiation phase (Additional file 3). Both, PPARs and TRs exert their activity partly by heterodimerization with RXR and they are known to play key roles in lipid mobilization, lipid degradation, fatty acid oxidation, and glucose metabolism [102]. In mammals, PPARs and TRs can crosstalk to regulate different cellular processes including adipogenesis [103]. The functional annotation analysis in the present study suggests that this might also be the case for rainbow trout adipocytes.

#### Fatty acid transport and oxidation during adipocyte maturation

**Fig. 7:**
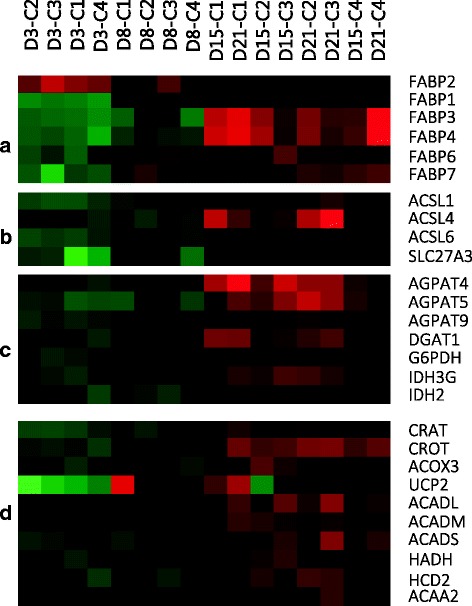
Selected genes involved in lipid metabolism. **a** Fatty acid transporters; **b** Acyl-CoA synthases; **c** Genes coding proteins involved in the synthesis of lipids; **d** Genes involved in energy production

The expression of proteins involved in fatty acid transport were mainly expressed during differentiation. We observed a high representation from the fatty acid binding protein family (FABPs) (Fig. 7a). Only FABP2 was up-regulated during proliferation, whilst FABP1, FABP3, FABP4 (which is predominantly expressed in mammalian adipose tissue) [104], FABP6 and FABP7 were all up-regulated during differentiation. The increasing levels of FABPs point out to the mobilization of fatty acids to mitochondria and peroxisomes for energy production. In mammals, FABPs are recognized as important signalling molecules with effects not only on the systemic energy metabolism but also on inflammatory processes [105]. In this sense, FABP4 acts as an adipokine regulating adipocyte and macrophage interactions during inflammation [106]. FABP3 transcripts have been predominantly detected in subcutaneous adipocytes [107] and intra-peritoneal adipose tissue of rainbow trout [23]. The high presence of FABPs suggests an important role for this family of proteins during the maturation process of fish adipocytes. In mammals, it has been reported that some FABPs significantly cooperate with PPARs [108]. Further studies focusing on the mechanisms of action of these proteins should be performed in fish in order to determine their function in the adipocyte biology.

After transport, fatty acids can be activated to the respective acyl-CoA esters by one of the acyl-CoA synthetases. In this sense, we found a strong enrichment of genes encoding several members of long chain fatty acid acyl-CoA synthetases in the differentiation cluster (Fig. 7b), such as ACSL1, ACSL4 and ACSL6, as well as the long-chain fatty acid transport protein 3 (SLC27A3). The presence of different ACSL isoforms might be essential for adipocyte function since they assure the channelling of fatty acids towards degradation or lipid biosynthesis depending on the energetic status of the cell.

A salient feature of the differentiation cluster was the presence of many genes encoding enzymes responsible of catalysing different steps along the triglyceride biosynthetic pathway (Fig. 7c). Such is the case for several members of the 1-acylglycerol-3-phosphate O-acyltransferase family (AGPAT4, AGPAT5 and AGPAT9) and the diacylglycerol O-acyltransferase 1 (DGAT1). In addition, genes involved in the production of reducing power for the synthesis of lipids, such as glucose-6 phosphate dehydrogenase (G6PDH) and isocitrate dehydrogenase (ICDH) were also present.

The activation of mechanisms responsible of providing energy to the adipocyte were also evidenced during the last stages of culture development (Fig. 7d). In this sense, proteins involved in the oxidation of fatty acids were present in the differentiation cluster, such as carnitine O-acetyltransferase (CRAT) and carnitine O-octanoyltransferase (CROT). These two enzymes convert the end products of the peroxisomal β-oxidation (C8 or C6-CoA) to acylcarnitines so they can be transported out of the peroxisomes and be further oxidized to acetyl-CoA in the mitochondria. We also found up-regulated during differentiation the expression of peroxisomal acyl-coenzyme A oxidase 3 (ACOX3), which is involved in the desaturation of 2-methyl branched fatty acids in peroxisomes. The expression of ACOX3 has been linked to a possible pathway for metabolism of phytanic acid and pristanic acid in peroxisomes in white adipose tissue [109]. In mammals it is known that β-oxidation of fatty acids can take place in both, mitochondria and peroxisomes, serving different functions in the cell [110]. The transcript abundance of other protein involved in lipid oxidation, like the mitochondrial uncoupling protein 2 (UCP2), was increased from day 8. This protein reduces the ATP yield and may facilitate the oxidation of fatty acids [111]. Many other genes involved in mitochondrial fatty acid beta-oxidation were present, such as acyl-CoA dehydrogenase, long chain (ACADL), acyl-CoA dehydrogenase, C-4 to C-12 straight chain (ACADM), acyl-CoA dehydrogenase, C-2 to C-3 short chain (ACADS), hydroxyacyl-CoA dehydrogenase (HADH), 3-hydroxyacyl-CoA dehydrogenase type-2 (HCD2) and acetyl-CoA acyltransferase 2 (ACAA2) among many others.

Overall, according to the functional characteristics of the mature adipocyte, genes involved in fatty acid transport, lipid synthesis and oxidation were the most relevant in late differentiation phase. The activation of diverse genes with redundant functions might be directed to provide different mechanisms to accomplish and ensure the proper function of mature differentiated adipocytes.

#### Lipid droplet formation

**Fig. 8:**
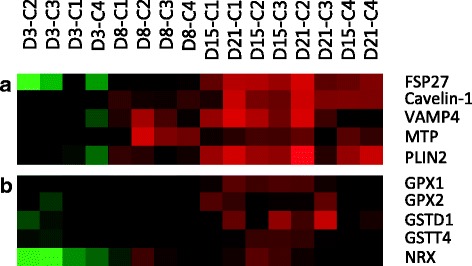
Selected genes highlighted during the terminal phase of adipocyte development. **a** Lipid droplet formation; **b** Antioxidant system

Mature white adipocytes are characterized by the presence of a large unilocular lipid droplet that occupies the majority of the cell. A critical aspect of adipocyte development is the formation and expansion of such lipid droplet within the cell. The storage of triglycerides within the lipid droplet allows the correct expansion of the adipose tissue while preventing lipotoxicity in other organs [112]. Therefore, lipid droplets are considered to be crucial organelles in the regulation of energy homeostasis and in the prevention of insulin resistance [113]. In the differentiation cluster we found up-regulated some of the well-known structural proteins that constitute the surface of the lipid droplets (Fig. 8a). Such is the case of the fat-specific protein 27/Cidec (FSP27) and Cavelin-1. Even though these proteins are not considered to be essential for adipocyte differentiation, their key role in lipid droplet formation has been recognized and thus also, in the acquisition of the adipocyte phenotype [114, 115]. Apart from their importance as structural proteins they have been described to play an important role in insulin signalling [46, 113]. Moreover, we also found highly expressed transcripts of the vesicle-associated membrane protein 4 (VAMP4), a member of the SNARE family known to be involved in the growth of lipid droplets via fusion [116]. Another protein that has been recently associated to lipid droplets, the microsomal triglyceride transfer protein (MTP), was also present in the differentiation cluster. This protein, which is known to be essential for lipid transport, has been reported to be present in the same droplets as perilipin 2 (PLIN2) [117]. Our results showed that PLIN2 exhibited the same pattern of expression as MTP, with significantly higher abundance of transcripts present during the differentiation phase of rainbow trout adipocytes. The study of specific mechanisms concerning the formation and turnover of lipid droplets would be of high value in order to elucidate the function of different associated proteins in lipid droplet biology in fish adipocytes.

#### Redox homeostasis

Adipocyte expansion and consequently lipid droplet growth, represents a healthy way of disposing excess of fat in the form of neutral lipids. Even though a continuous or extreme expansion can represent a challenge to adipocytes leading to the production of reactive oxygen species and endoplasmic reticulum stress [112], a moderate generation of reactive oxygen species has been described as a factor promoting adipocyte differentiation [118]. Therefore, the control of redox homeostasis in the intracellular environment is key for adipogenesis to progress. In line with this, we identified few genes encoding antioxidant enzymes (Fig. 8b), such as glutathione peroxidase 1 and 2 (GPX1 and GPX2), glutathione S-transferase 1, isoform D (GSTD1) and glutathione S-transferase theta-4 (GSTT4), all of them induced during the lipid-loading phase. The modulation of intracellular glutathione has been reported to affect adipogenesis in 3 T3-L1 cells [119]. In addition, the importance of the activation of the gluthatione-based antioxidant system in Atlantic salmon adipocytes has been recently highlighted [14]. Likewise, nucleoredoxin (NRX), a member of the thioredoxin family of proteins known to control redox homeostasis in the cell, followed the same regulation in rainbow trout adipocytes. Interestingly, NRX has recently been described as a novel pro-adipogenic factor [120].

### Temporal profile analysis of mRNA (qPCR) and protein expression

**Fig. 9:**
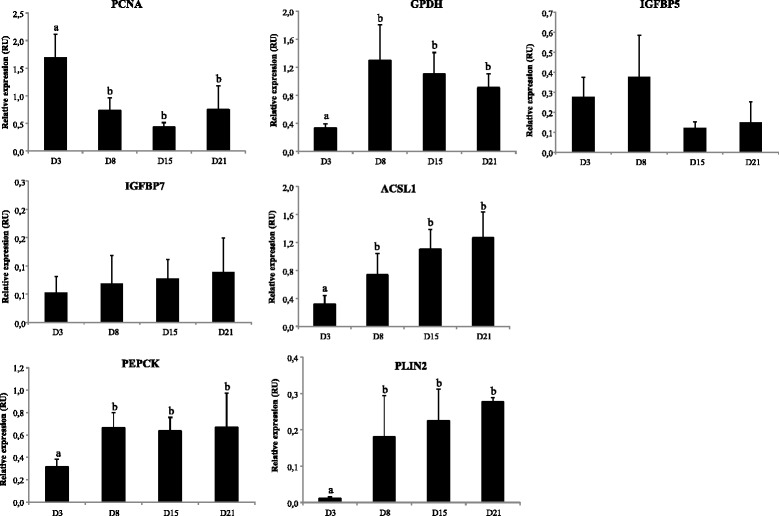
Relative changes in transcript levels of selected genes during trout adipocyte differentiation (days 3, 8, 15 and 21) assessed by qPCR. Transcript abundance is presented as fold change ± S.E.M (*n* = 4) using 18S as a reference gene (delta-delta method). (RU): relative units. Values not sharing letters are significantly different (*P* < 0.05, ANOVA followed by Tukey’s test)

**Fig. 10:**
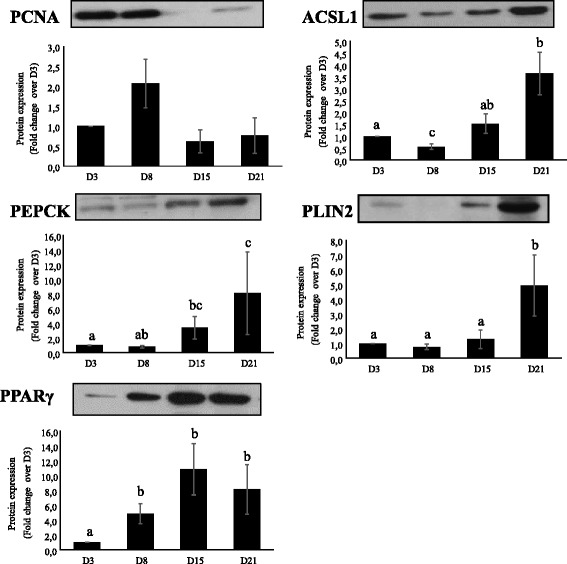
Relative changes in content of selected proteins during rainbow trout adipocyte differentiation (days 3, 8, 15 and 21) assessed by Western blot analysis. Protein abundance (*n* = 4) was performed as described in the materials and methods section. A representative blot is shown

The expression profile along the culture of some selected genes was tested using qPCR (Fig. 9). The genes were selected on the basis of showing differential expression, their novelty in fish or their relevance and implication in the already described mammalian adipogenic process. Despite of the activation of ribosome biogenesis during the early phase of adipocyte development, 18S was found to be the most stable reference gene, suggesting that some regulatory proteins involved in this process other than 18S might be differentially regulated. Additionally, and despite the potential confounding effect of normalizing to a reference gene, both the relative abundance of the target genes prior to normalization and the transcriptomic data supports the presented values. The levels of protein of some of the selected genes were also tested, in order to assess whether the protein encoded by a specific mRNA and its expression showed a similar profile (Fig. 10). Both qPCR and Western blot analysis reinforced the data obtained in the microarray analysis. The high proliferative capacity of the cells during the early stages determined by the gene expression pattern of proliferating cell nuclear antigen (PCNA), which had its highest peak in the transcriptome at day 3 of rainbow trout adipocyte development, was further reinforced by qPCR and Western blot analysis. Likewise, the maturation state of the cells was confirmed by both, microarray and qPCR measures of glycerol-3-phosphate dehydrogenase (GPDH). In addition, the transcript abundance of two IGFBPs aforementioned, IGFBP5 and IGFBP7, were analyzed by qPCR. Despite of the lack of significance, IGFBP5 followed the same pattern as the one observed in the microarray, while the mRNA of IGFBP7 remained fairly static along the culture according to qPCR results. Moreover, two genes representing the development of lipid and carbohydrate metabolism, ACSL1 and PEPCK respectively, were assessed as well by qPCR and Western blot, confirming the specialization of the cells towards mature adipocytes showing higher levels of expression at later days and validating the microarray results. In addition, PLIN2 was also evaluated through all three techniques, exhibiting a higher abundance during the differentiation phase and suggesting thus the involvement of this structural protein in the maturation process of our cell system. It should be noted that, as far as we know, no member from the perilipin family has been characterized in fish until now. Therefore, the expression and function of this gene deserves further studies. On the other hand, the protein levels of PPARγ, which is considered the master regulator of adipogenesis, were significantly increased from day 8 and maintained up to day 21. Therefore, confirming the relevance of the PPAR signaling pathway during the differentiation phase revealed by the transcriptomic analysis and previous studies [5].

Likewise, as we have described in the present paper, many other key molecules known to be involved in the adipogenic process of mammalian preadipocyte cell lines, also changed in the same direction in our culture system. Therefore, these kinds of approaches can be seen as an extra validation of the microarray results.

## Conclusions

This study was performed in order to gain a comprehensive view of the regulatory mechanisms involved in the process of fat cell development in fish. Our cell system presents a high similarity with the already described process of adipocyte differentiation in mammalian models, with abundance of several of the pro-adipogenic transcription factors and many of the mature adipocyte related genes. As it is evidenced by the large number of genes transcriptionally regulated in this study, fish adipogenesis is a complex and tightly coordinated process.

In mammals, adipocyte dysregulation has been linked to the development of metabolic diseases and therefore remarkable efforts are being done to understand the intricacies of adipogenesis. Many aspects concerning the biology of fish adipose tissue and its contribution to the balance of the overall energy homeostasis remain unexplored. This study provides an important base for further research. However, functional approaches will be needed in order to clarify the role of the genes highlighted in the present work.

## Additional files

Additional file 1: Significant biological functions associated with the proliferative phase of adipogenesis determined by IPA software. (XLSX 13 kb)

Additional file 2: List of genes from the highlighted biological functions associated with the proliferative phase of adipogenesis determined by IPA software. (XLSX 104 kb)

Additional file 3: Significant biological functions associated with the differentiation phase of adipogenesis determined by IPA software. (XLSX 14 kb)

Additional file 4: List of genes from the highlighted biological functions associated with the differentiation phase of adipogenesis determined by IPA software. (XLSX 47 kb)

## Abbreviations

ACSL: Long-chain acyl-CoA synthetase
C/EBP: CCAAT-enhancer binding protein
GPDH: Glycerol-3-phosphate dehydrogenase
IGFBP: Insulin-like growth factor binding protein
IPA: Ingenuity pathway analysis
MSCs: Mesenchymal stem cells
PCNA: Proliferating cell nuclear antigen
PEPCK: Phosphoenolpyruvate carboxykinase
PLIN2: Perilipin 2
PPARγ: Peroxisome proliferator activated receptor γ
